# The First Outbreak of Dengue Fever in Greater Darfur, Western Sudan

**DOI:** 10.3390/tropicalmed4010043

**Published:** 2019-03-01

**Authors:** Ayman Ahmed, Adel Elduma, Babiker Magboul, Tarig Higazi, Yousif Ali

**Affiliations:** 1 Institute of Endemic Diseases, University of Khartoum, Khartoum 11111, Sudan; 2 Department of Vector Biology, Liverpool School of Tropical Medicine (LSTM), Liverpool L3 5QA, UK; 3 Epidemiology Department, National Public Health Laboratory, Khartoum 11111, Sudan; dumanet@gmail.com; 4 Health Emergencies and Epidemics Control Directorate, Sudan Federal Ministry of Health, Khartoum 11111, Sudan; babkerali@yahoo.com (B.M.); yousif.health@gmail.com (Y.A.); 5 Department of Biological Sciences, Ohio University, Zanesville, OH 45701, USA; higazi@ohio.edu

**Keywords:** dengue, outbreak, emergence, West Nile virus (WNV), hemorrhagic fever, Crimean–Congo hemorrhagic fever (CCHF), epidemic, *Aedes aegypti*, Greater Darfur, Sudan

## Abstract

Dengue virus (DENV) is an arthropod-borne virus (arbovirus) transmitted by the *Aedes* mosquitoes, mainly *Aedes aegypti*. Dengue fever is a rapidly growing disease with expanding geographical distribution worldwide. We investigated a high number of non-malaria febrile cases reported to health clinics in refugee camps in the five states of Darfur between August 2015 and March 2016. The clinical presentation of cases and case definition criteria suggested involvement of one or more arboviral hemorrhagic fevers. Out of 560 suspected cases, we collected and analyzed 204 blood samples and serologically positive samples were confirmed by PCR. We identified 32 (15.7%) dengue viral infections, six West Nile virus infections, and three Crimean–Congo viral infections. Dengue infections were found in four out of the five Darfur states. We reported the first dengue fever outbreak in the Darfur region. Our results highlight the need for public health education and further molecular, phylogenetic, and entomological investigations for a better understanding of the disease transmission and the associated risk factors in the region.

## 1. Introduction

Dengue fever (DF) is a mosquito-borne viral disease caused by one of four closely-related dengue virus serotypes (DENV1–4) of the genus *Flavivirus* and family Flaviviridae and it is mainly transmitted by *Aedes aegypti* with other species of *Aedes* mosquito involved [1,2]. Dengue infection has different clinical presentations ranged from a self-limiting flu-like illness to the fatal severe form of dengue hemorrhagic fever or dengue shock syndrome [1,3]. Dengue is a rapidly expanding arboviral infection currently present in at least 128 countries with an estimated 390 million dengue infections annually and 3.97 billion people at risk of infection [4,5,6].

Dengue virus transmission is influenced by several factors, including climate change [7], the global trade, human population dynamics, and international travel, that facilitate the spread of the vectors and introduction of the DENV into new areas [8,9,10,11,12]. Unplanned urbanization and high human population density play a major role in the DENV transmission and outbreaks [13,14,15].

DF is a global public health problem with common epidemics in the tropical countries. DF incidences are greatly underestimated because most dengue infections are self-limiting, inapparent cases that usually go unreported [3,4]. The unnoticeable, persistent transmission of the dengue virus usually results in the emergence of epidemics of different scales which are mainly influenced by the human population density, susceptibility, and previous exposure to DENV and the density of the mosquito vectors [1,16,17]. In addition, armed conflicts and living in a humanitarian setting renders communities more vulnerable to infectious diseases, including DF [18].

In Sudan, DF is considered a major public health issue in the eastern region of the country, where it has been reported since 1908 [19], with endemicity and frequent outbreaks in the coastal and sub-coastal areas of the Red Sea and Kassala states [20,21,22,23,24]. Yellow fever and Crimean–Congo hemorrhagic fever are endemic in Darfur and the area has recently suffered from one of the worst yellow fever epidemics worldwide [25,26,27]. The outbreak of yellow fever was influenced by the living condition in a humanitarian setting that favored the establishment of *Ae. aegypti*, the same main vector of DF [1,18]. In this communication, we report the first outbreak of dengue and dengue hemorrhagic fever along with West Nile virus and Crimean–Congo hemorhagic fever infections in the greater Darfur region, Western Sudan.

## 2. Materials and Methods 

### 2.1. Study Area

**Figure 1:**
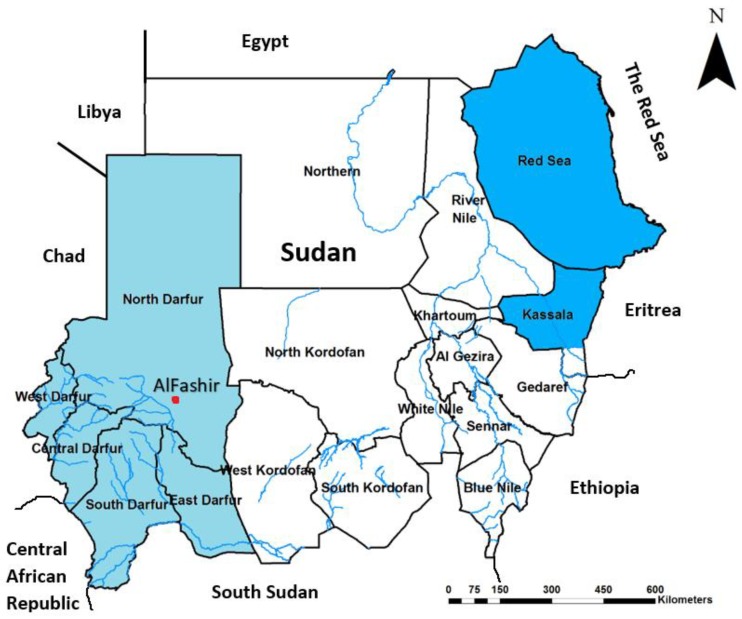
Map of Sudan showing the study area of Greater Darfur (in the light blue color, and dengue fever endemic areas in Sudan (the darker blue).

This health facility-based cross-sectional study was conducted in 29 health facilities, mainly in refugee camps throughout Darfur region. Darfur is a vast region of 5 federal states located in western Sudan, along the borders with Libya, Chad, Central African Republic, and South Sudan (Figure 1), with a total area of 493,180 km^2^ and population of 9,241,369 individuals. It is located between 14.3783° N, 24.9042° E and in mainly desert and semi-arid area. Since 2003, the area has been affected by an armed conflict and civil unrest that has led to massive internal displacement and immigration [28]. 

### 2.2. The Outbreak Investigation

An unusually high number of cases of non-malaria febrile illness were reported in the region in August 2015. Following a well-established case definition (Sudan Federal Ministry of Health) based on the clinical presentation, we identified suspected cases of hemorrhagic fever presenting to the health clinics of 29 refugee-camps in East, West, South, North, and Central Darfur states (Figure 1) between August 29 2015 and February 16 2016. Blood samples were collected from patients as soon as they were identified as a suspected case and tested for the major arboviral infectious agents of dengue fever (DF), yellow fever (YF), Rift Valley fever (RVF), Crimean–Congo Hemorrhagic Fever (CCHF), West Nile virus (WNV), chikungunya virus (CHIKV), and Zika virus (ZIKV). Arboviral-specific enzyme-linked immunosorbent assays (IgM capture ELISA) were performed using commercially available kits and following manufacturer’s instructions (Panbio, Inverness Medical Innovations Australia Pty Ltd, Brisbane, Australia). Serologically positive samples were confirmed by using commercially available Real Time RT-PCR Kits (Shnaghai ZJ Bio-Tech Co.Ltd, Shanghai, China) following manufacturer’s guidelines. Due to limited blood samples and resources, using blood samples left from previous analyses, up to 5 DENV positive samples were pooled together for the DENV serotype analysis. All serological and molecular assays were done at the Sudan National Public Health Laboratory. This study has been done using clinical samples and data obtained during the outbreak and all personal identifiers have been excluded.

## 3. Results

**Figure 2:**
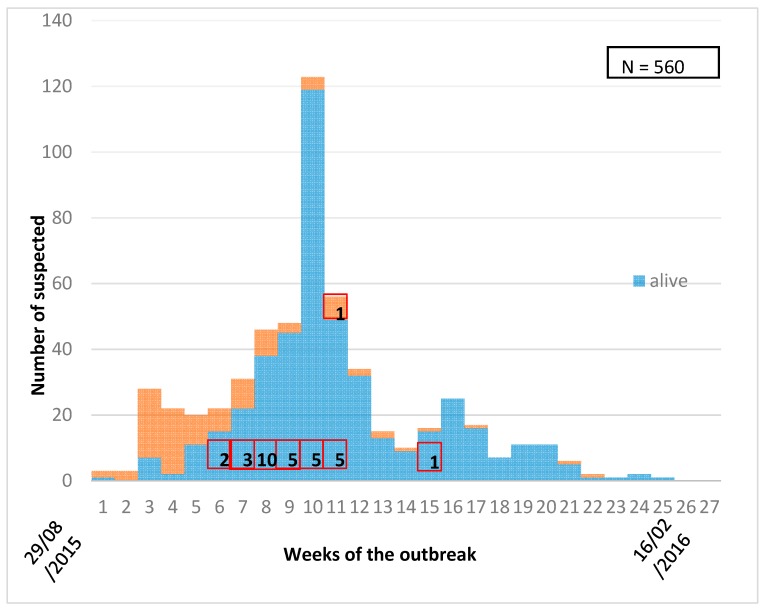
Epidemic curve of the first dengue fever outbreak in Greater Darfur showing the development of the outbreak and case fatality from August 2015 to February 2016. Number of DF confirmed cases per epidemic week showed in red boxes.

**Figure 3:**
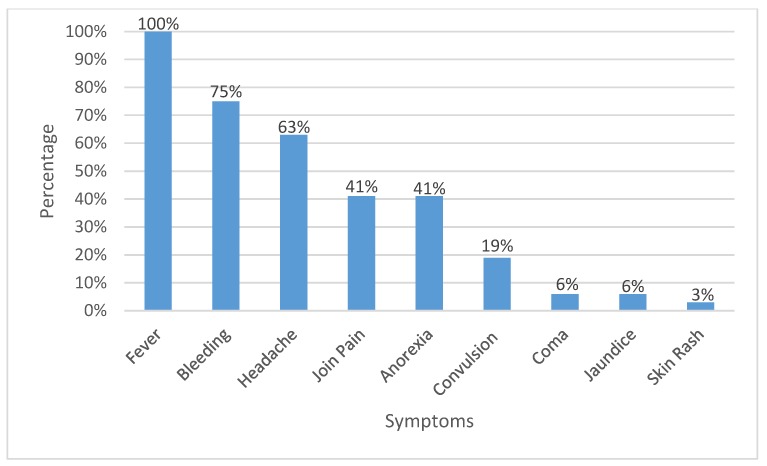
Clinical presentations of the confirmed cases of dengue fever.

We identified 560 suspected cases of hemorrhagic fever from 29 localities across the five states of Darfur during the study period and the majority of the cases were from the West Darfur state (Table 1). The first suspected cases appeared on 29 August 2015 and the last one was reported on 16 February 2016, with the peak of the outbreak in November (Figure 2). The fatality rate among the suspected cases was 18.2%, but only one confirmed case of dengue died. The clinical presentation of the dengue infections was generally severe, with all cases presenting with fever. The majority presented with bleeding and headache and less than half of cases with joint pain and anorexia (Figure 3). Nearly two-thirds of the suspected cases refused to donate blood samples (Table 1).

Molecular analysis confirmed 32 positive cases of dengue fever out of the 204 (15.7%) from 11 localities in the Darfur area with 24 (75%) cases from the West Darfur state. Six (2.9%) cases of West Nile virus (WNV) were detected in samples from the states of West and North Darfur and three (1.5%) cases of Crimean–Congo hemorrhagic fever (CCHF) were identified in East Darfur (Table 1). No cases of Zika virus (ZIKV), yellow fever (YF), chikungunya virus (CHIKV), or Rift Valley fever (RVF) were detected (Table 1). Most of the dengue infections (72%) were in children and young adults under 25 years old, while all CCHF cases were adults (Table 2). We found no association between infection and gender. Limited serotype analysis of DENV serotypes revealed that both DENV-1 and DENV-3 exist in the area.

## 4. Discussion

In this communication, we investigated the first outbreak of dengue fever in the Darfur area and we confirmed the presence of DENV in all states of Darfur, except East Darfur. We believe the prevalence of dengue fever cases might be higher than reported here because of the passive nature of our survey and reliance on patients’ presentation at the health facilities, imbalance in the blood sample donation between areas, and the refusal rate for blood donation. The DENV might be present in East Darfur, but we failed to detect it because most DENV infections are mild or asymptomatic [3]. The majority (75%) of the detected DENV infections presented with bleeding, suggesting the involvement of dengue hemorrhagic fever, one of the severe forms of the disease according to the guidelines of the World Health Organization [2]. Dengue fever in Sudan has been localized to eastern Sudan [20,21,24], with frequent epidemics in the area since the early 1900s [19,23,27]. Darfur has a recent history of yellow fever epidemics [25,29], but DENV has only been detected in a few cases in AlFashir, the capital city of North Darfur, for the first time in 2014. 

Compared to dengue fever, similarly high infections of yellow fever among children and young adults have recently been reported in the same area. Both arboviral infections are transmitted by the same mosquito vector, *Ae. aegypti,* suggesting similarity in the behavior of either human, vector, or both [29]. More detailed studies are needed to be address the role of the mosquito vector of arboviruses in Darfur. Unlike previous studies of arboviral outbreaks in the area, we did not notice any association between infection and gender [29].

The severe clinical presentations of dengue fever cases during this outbreak seems to suggest a lack of previous exposure, but it could also be attributed to the co-circulation of two serotypes of dengue virus 1 and 3, since a secondary infection with a different serotype of the virus is a risk factor for developing severe disease [1]. Our limited serotype analysis showed both DENV-1 and -3 in the area and did not exclude others, even though DENV-2 has been recently identified in Kassala state, East Sudan [30]. In addition, our serotype analysis did not address the issue of co-infection with multiple serotypes. The notably high negative results could be due to collection of the blood samples before the development of a detectable immune response (antibodies). The protocol of the Sudan National Public Health Laboratory is to use serological tests to screen the blood samples for infections, followed by using RT-PCR assays to confirm the serologically positive cases [31]. The high mortality rate in the first weeks of the outbreak might be attributed to the start of treatment before confirmation of DENV infection. Most of these cases were treated as severe malaria cases, which is a common confusion in resource-limited settings [25].

Greater Darfur has suffered civil war and massive population displacement that led most people to live in very populated refugee camps with limited basic services [18]. Such conditions increased the vulnerability of the population to the risk of infectious diseases, including dengue fever and other arboviral infections, and made the health system very fragile. The arrival of the UNAMID (The African Union—United Nations Hybrid Operation in Darfur) peacekeeping force to Darfur is a possible scenario for the introduction of the DENV into the area, as some of those troops are coming from dengue fever endemic areas [18,27]. Another possible scenario for introducing DENV into the area is through immigrants from neighboring countries with endemic dengue fever, who came to work in the local gold mines [28]. Later entomological surveys showed *Ae. aegypti* to be the dominant mosquito in the area, with an incidence of up to 86% in manmade water containers (unpublished data), which refugees use to store water. This created typical breeding sites for the *Ae. aegypti* mosquito. This situation has most likely contributed to the epidemic of yellow fever in the same area in 2012 [8,29,31].

One of the limitations of this study is the refusal of blood sample donation for diagnosis that has affected our sample size. Such refusal has been noted previously in the area [31] and requires public health campaigns to educate the local population about these infectious agents and encourage health care seeking behavioral change. Another limitation was our inability to run a full serotype analysis of DENV and investigating the co-infection possibility. In addition, this survey has been based on passive surveillance and only detected severe cases from healthcare centers. Further entomological, molecular, and phylogenetic investigations are urgently needed to improve our understanding of the risk factors influencing the emergence and outbreaks of dengue fever in the area. Furthermore, we highlight the need to improve the health and living conditions of people in refugee camps and the need for preventive measures against new and endemic arboviral diseases.

## Figures and Tables

**Table 1:** Number of hemorrhagic fever suspected cases, ratio of blood samples obtained and lab results per state of Greater Darfur from 29 August 2015 to 16 February 2016.

State	Suspected Cases	Samples Collected (Percentage)	DENV	WNV	CCHFV	Negative
Central Darfur	74	55 (74%)	5	0	0	50
East Darfur	26	22 (85%)	0	0	3	19
North Darfur	128	27 (21%)	1	1	0	25
South Darfur	20	15 (75%)	2	0	0	13
West Darfur	312	85 (27%)	24	5	0	56
Total	560	204 (36%)	32	6	3	163

DENV: dengue virus; WNV: West Nile virus; CCHFV: Crimean–Congo hemorrhagic fever virus.

**Table 2:** Age and sex ratio of confirmed arboviral infections.

Age Group	DF	WNV	CCHF	F:M
0–10 years	12	0	0	1:2
11–25 years	11	5	0	1.3:1
26–45 years	8	1	3	1:1
Elder than 46 years	1	0	0	1:0
Total	32	6	3	-

DF: dengue fever; WNV: West Nile virus; CCHFV: Crimean–Congo hemorrhagic fever. F:M = Female to Male ratio.

## References

[1] Simmons C.P., Farrar J.J., van Vinh Chau N., Wills B. (2012). Dengue. N. Engl. J. Med..

[2] World Health Organization (2009). Dengue Guidelines for Diagnosis, Treatment, Prevention and Control: New Edition.

[3] Endy T.P., Anderson K.B., Nisalak A., Yoon I.-K., Green S., Rothman A.L., Thomas S.J., Jarman R.G., Libraty D.H., Gibbons R.V. (2011). Determinants of Inapparent and Symptomatic Dengue Infection in a Prospective Study of Primary School Children in Kamphaeng Phet, Thailand. PLoS Negl. Trop. Dis..

[4] Bhatt S., Gething P.W., Brady O.J., Messina J.P., Farlow A.W., Moyes C.L., Drake J.M., Brownstein J.S., Hoen A.G., Sankoh O. (2013). The global distribution and burden of dengue. Nature.

[5] Brady O.J., Gething P.W., Bhatt S., Messina J.P., Brownstein J.S., Hoen A.G., Moyes C.L., Farlow A.W., Scott T.W., Hay S.I. (2012). Refining the global spatial limits of dengue virus transmission by evidence-based consensus. PLoS Negl. Trop. Dis..

[6] Guzman M.G., Halstead S.B., Artsob H., Buchy P., Farrar J., Gubler D.J., Hunsperger E., Kroeger A., Margolis H.S., Martínez E. (2010). Dengue: A continuing global threat. Nat. Rev. Microbiol..

[7] Van Kleef E., Bambrick H., Hales S. The geographic distribution of dengue fever and the potential influence of global climate change. http://eprints.qut.edu.au/103224/.

[8] Tatem A.J., Hay S.I., Rogers D.J. (2006). Global traffic and disease vector dispersal. Proc. Natl. Acad. Sci. USA.

[9] Cummings D.A.T., Irizarry R.A., Huang N.E., Endy T.P., Nisalak A., Ungchusak K., Burke D.S. (2004). Travelling waves in the occurrence of dengue haemorrhagic fever in Thailand. Nature.

[10] Wilder-Smith A., Gubler D.J. (2008). Geographic Expansion of Dengue: The Impact of International Travel. Med. Clin..

[11] Wesolowski A., Qureshi T., Boni M.F., Sundsøy P.R., Johansson M.A., Rasheed S.B., Engø-Monsen K., Buckee C.O. (2015). Impact of human mobility on the emergence of dengue epidemics in Pakistan. Proc. Natl. Acad. Sci. USA.

[12] Stoddard S.T., Morrison A.C., Vazquez-Prokopec G.M., Soldan V.P., Kochel T.J., Kitron U., Elder J.P., Scott T.W. (2009). The Role of Human Movement in the Transmission of Vector-Borne Pathogens. PLoS Negl. Trop. Dis..

[13] Gubler D.J. (2002). Epidemic dengue/dengue hemorrhagic fever as a public health, social and economic problem in the 21st century. Trends Microbiol..

[14] Kendall C., Hudelson P., Leontsini E., Winch P., Lloyd L., Cruz F. (1991). Urbanization, Dengue, and the Health Transition: Anthropological Contributions to International Health. Med. Anthropol. Q..

[15] Gubler D.J. (2011). Dengue, Urbanization and Globalization: The Unholy Trinity of the 21st Century. Trop. Med. Health.

[16] Brathwaite Dick O., San Martín J.L., Montoya R.H., del Diego J., Zambrano B., Dayan G.H. (2012). The History of Dengue Outbreaks in the Americas. Am. J. Trop. Med. Hyg..

[17] Brady O.J., Smith D.L., Scott T.W., Hay S.I. (2015). Dengue disease outbreak definitions are implicitly variable. Epidemics.

[18] Gayer M., Legros D., Formenty P., Connolly M.A. (2007). Conflict and Emerging Infectious Diseases. Emerg. Infect. Dis..

[19] Balfour A., Archibald R.G. (1908). Review of Some of the Recent Advances in Tropical Medicine, Hygiene and Tropical Veterinary Science.

[20] Soghaier M.A., Himatt S., Osman K.E., Okoued S.I., Seidahmed O.E., Beatty M.E., Elmusharaf K., Khogali J., Shingrai N.H., Elmangory M.M. (2015). Cross-sectional community-based study of the socio-demographic factors associated with the prevalence of dengue in the eastern part of Sudan in 2011. BMC Public Health.

[21] Seidahmed O.M., Hassan S.A., Soghaier M.A., Siam H.A., Ahmed F.T., Elkarsany M.M., Sulaiman S.M. (2012). Spatial and temporal patterns of dengue transmission along a Red Sea coastline: A longitudinal entomological and serological survey in Port Sudan city. PLoS Negl. Trop. Dis..

[22] Abdallah T.M., Ali A.A.A., Karsany M.S., Adam I. (2012). Epidemiology of dengue infections in Kassala, Eastern Sudan. J. Med. Virol..

[23] Malik A., Earhart K., Mohareb E., Saad M., Saeed M., Ageep A., Soliman A. (2011). Dengue hemorrhagic fever outbreak in children in Port Sudan. J. Infect. Public Health.

[24] Adam I., Jumaa A.M., Elbashir H.M., Karsany M.S. (2010). Maternal and perinatal outcomes of dengue in PortSudan, Eastern Sudan. Virol. J..

[25] Markoff L. (2013). Yellow Fever Outbreak in Sudan. N. Engl. J. Med..

[26] Ibrahim A.M., Adam I.A., Osman B.T., Aradaib I.E. (2015). Epidemiological survey of Crimean Congo hemorrhagic fever virus in cattle in East Darfur State, Sudan. Ticks Tick-Borne Dis..

[27] Seidahmed O.M.E., Siam H.A.M., Soghaier M.A., Abubakr M., Osman H.A., Abd Elrhman L.S., Elmagbol B., Velayudhan R. (2012). Dengue Vector Control and Surveillance during a Major Outbreak in a Coastal Red Sea Area in Sudan. http://www.who.int/iris/handle/10665/118472.

[28] Olsson O. (2010). After Janjaweed? Socioeconomic Impacts of the Conflict in Darfur. World Bank Econ. Rev..

[29] Ahmed S.S., Soghaier M.A., Mohammed S., Khogali H.S., Osman M.M., Abdalla A.M. (2016). Concomitant outbreaks of yellow fever and hepatitis E virus in Darfur States, Sudan, 2012. J. Infect. Dev. Ctries.

[30] Hamid Z., Hamid T., Alsedig K., Abdallah T., Elaagip A., Ahmed A., Khalid F., Abdel Hamid M. (2019). Molecular Investigation of Dengue virus serotype 2 Circulation in Kassala State, Sudan. Jpn. J. Infect. Dis..

[31] Soghaier M.A., Hagar A., Abbas M.A., Elmangory M.M., Eltahir K.M., Sall A.A. (2013). Yellow Fever outbreak in Darfur, Sudan in October 2012; the initial outbreak investigation report. J. Infect. Public Health.

